# Consonant Error Profiles and Short-Term Memory Deficits in Chinese School-Age Children with Speech Sound Disorders

**DOI:** 10.3390/bs16040540

**Published:** 2026-04-05

**Authors:** Qi Xu, Nan Peng, Xihan Li, Lei Wang, Haifeng Duan, Cuijuan Xu, Xi Wang, Bo Zhou, Jianhong Wang, Lin Wang

**Affiliations:** 1 Department of Child Health Care, Capital Center for Children’s Health, Capital Medical University, Capital Institute of Pediatrics, Beijing 100020, China; cookie7826@mail.ccmu.edu.cn (Q.X.); b2023032008@pumc.edu.cn (N.P.); lgs1003455923@163.com (L.W.); xcjlt@mail.ccmu.edu.cn (C.X.); 15910683272@mail.ccmu.edu.cn (X.W.); bobo-0207@mail.ccmu.edu.cn (B.Z.); 2 Capital Institute of Pediatrics, Chinese Academy of Medical Sciences & Peking Union Medical College, Beijing 100020, China; 3 School of Public Health, Capital Medical University, Beijing 100069, China; 2218031@mail.ccmu.edu.cn; 4 College of Chinese Minority Languages and Literature, Minzu University of China, Beijing 100081, China; haifengduan@muc.edu.cn

**Keywords:** speech sound disorder, consonant accuracy, short-term memory, backward digit span

## Abstract

Speech sound disorder (SSD) is common in childhood and can persist, adversely affecting language, literacy, and social functioning. Yet consonant error patterns in school-age children, particularly in non-English-speaking populations, remain insufficiently characterized. Short-term memory (STM) supports phonological processing and speech learning, but its relationship with SSD severity in school-age children is not well established. This study profiles consonant errors and short-term memory in school-age Chinese children with SSD and examines short-term memory correlates and predictors of disorder severity to inform targeted interventions. A total of 142 Mandarin-speaking school-age children with SSD were recruited. For the short-term memory analyses, we randomly selected 70 children with SSD and recruited 70 typically developing controls. Speech was assessed using a word-level picture-naming task to derive consonant accuracy and characterize error types/patterns, and short-term memory was measured with the WISC-IV Digit Span (forward and backward). Substitutions predominated for most consonants, and individual phonemes often exhibited co-occurring error patterns. In addition, school-age children with SSD showed significantly poorer short-term memory than typically developing peers across multiple indices. Notably, backward digit span was positively associated with consonant accuracy and remained an independent predictor of consonant accuracy. These results advance our understanding of the mechanisms underlying SSD and provide an evidence-based rationale for future interventions that combine speech-focused therapy with cognitive training to enhance clinical outcomes.

## 1. Introduction

Speech sound disorder (SSD) is a common childhood communication disorder characterized by speech sound production that is inappropriate for a child’s age and developmental stage. Errors in one or more phonemes—either occurring in isolation or in combination—can result in inaccurate word productions and reduced speech intelligibility (Hearnshaw et al., 2019). The occurrence of SSD may be related to immaturity in the speech production system and/or the speech perception system (Tambyraja et al., 2020); it is not attributable to congenital or acquired disease, and affected children typically present with normal hearing and no structural abnormalities or motor dysfunction of the speech apparatus (Hearnshaw et al., 2019). In some children, speech sound errors resolve spontaneously with age. However, approximately 30–50% of children with SSD may develop persistent speech sound disorder, with negative consequences for language comprehension, expressive language, and social functioning (Lewis et al., 2025; Tambyraja et al., 2020). Weaknesses in speech processing tend to predispose affected children to comorbid learning difficulties including developmental dyslexia (Baddeley, 2003) and spelling impairment as they grow older; thus, SSD may exert substantial long-term impacts on academic achievement and subsequent employment outcomes (Harding et al., 2024). However, existing research on SSD in Chinese-speaking children has mainly focused on preschool-aged populations. In contrast, relatively little attention has been paid to school-age children with SSD. Moreover, few studies in Chinese-speaking populations have examined factors associated with SSD severity.

Short-term memory (STM), particularly phonological short-term memory, is a limited-capacity system that temporarily maintains speech-based representations over seconds and supports the online encoding, sequencing, and monitoring of phonemes during speech perception and production, making it especially relevant to children’s acquisition and stabilization of speech sound categories (Newbury et al., 2005). A growing body of evidence suggests that children with speech sound disorders (SSD) may present with STM deficits (Geronikou et al., 2023). Previous studies have also suggested that the severity of verbal short-term memory impairment is positively associated with the severity of SSD (Tambyraja et al., 2023). However, investigations examining the association between the severity of SSD in school-age children and STM remain limited.

STM deficits not only compromise children’s ability to retain and reproduce target speech sounds during imitation and practice, but may also indirectly impede lexical and grammatical development by reducing the stability of phonology–semantics mappings (Smith et al., 2024). Evidence further suggests that children with stronger STM capacity tend to show more efficient gains during speech intervention, whereas persistent STM limitations are associated with a higher risk of enduring speech sound difficulties (Minkina et al., 2017; Yasmin et al., 2022). Therefore, beyond characterizing speech error patterns in school-age children with SSD, clarifying their STM profiles and identifying STM-related factors linked to disorder severity may provide important insight into underlying mechanisms and help inform more comprehensive assessment and intervention planning.

Our study aims to characterize consonant error patterns and short-term memory profiles in school-age Chinese children with SSD, to determine how STM relates to severity of SSD, and to identify factors that predict the severity of speech impairment. By integrating these findings, we intend to create more precise and evidence-based intervention strategies for this population.

## 2. Materials and Methods

### 2.1. Participants Recruitment

For the investigation of consonant characteristics in children with SSD, a total of 142 school-age children diagnosed with SSD were recruited and assigned to the SSD group. All participants received their initial diagnosis at their first visit to the Speech and Language Clinic of the Capital Institute of Pediatrics between January 2021 and December 2023. Participants were divided into three age groups (6 to <7 years, 7 to <8 years, and ≥8 years) based on both developmental considerations and the actual sample distribution. The two younger groups were separated because consonant acquisition and speech development remain relatively dynamic during these years. In contrast, children older than 8 years were combined into one group because basic consonant acquisition is generally more stable after this stage, and further subdivision would have resulted in relatively small subgroup sizes and less reliable statistical comparisons.

To compare STM performance between children with SSD and typically developing peers, typically developing children who underwent routine health examinations at the same institute during the same period were invited to participate. However, only 70 typically developing children and their families agreed to complete the speech and STM assessments and were therefore included in the control group. To ensure group comparability in the STM analyses, 70 children were selected from the 142 children originally identified in the SSD group using a random number table for inclusion in the comparative analyses. Participants were recruited on the basis of the predefined inclusion and exclusion criteria and the availability of eligible children rather than through individual age- and sex-matching.

All enrolled children and their families were native Mandarin speakers and had normal developmental and intelligence assessments. This study was approved by the Ethics Committee of the Capital Institute of Pediatrics (SHERLL2021079), and written informed consent was obtained from all parents/guardians.

Inclusion criteria for the SSD group were as follows:

(1) A diagnosis of speech sound disorder according to the criteria of the Diagnostic and Statistical Manual of Mental Disorders, Fifth Edition (DSM-5) (Hearnshaw et al., 2019).
(2) No structural abnormalities or motor dysfunction of the speech apparatus, with normal hearing, normal intelligence, and typical language developmental trajectory and language abilities.
(3) Persistent symptoms of imprecise articulation for at least 36 months.
(4) Age of 6–16 years.
(5) Both the child and family were Mandarin speakers. Inclusion criteria for the control group were typically developing children aged 6–16 years.

Exclusion criteria for both groups included: orofacial malformations, hearing impairment, adverse perinatal history (preterm birth, intrauterine distress at birth, or neonatal asphyxia), and neurological disorders, psychiatric disorders, inherited metabolic diseases, or other neurodevelopmental conditions, including known language delay or language disorders.

### 2.2. Speech Assessment

A word-level picture-naming task was administered. Consonant error types and error patterns were recorded, and consonant accuracy was calculated. Consonant accuracy was defined as number of correct consonants/(number of correct consonants + number of incorrect consonants) × 100%. Severity was classified according to consonant accuracy as follows: <50% (severe), 50–65% (moderate-to-severe), 66–85% (mild-to-moderate), and 86–100% (mild) (Shriberg & Kwiatkowski, 1982).

### 2.3. Consonants and Their Error Classification

(1) Consonant classification: A total of 21 Mandarin consonants were included. According to place of articulation, these consonants were classified into six categories: labials ([p] b, [p^h^] p, [m] m, [f] f), alveolo-palatals ([tɕ] j, [tɕ^h^] q, [ɕ] x), velars ([k] g, [k^h^] k, [x] h), apical anterior ([ts] z, [ts^h^] c, [s] s), apical medial ([t] d, [t^h^] t, [n] n, [l] l), and apical posterior ([ʈʂ] zh, [ʈʂ^h^] ch, [ʂ] sh, [ɻ] r).
(2) Classification of consonant error types: Errors in which a target consonant was replaced by another consonant differing in place of articulation, or by a consonant produced with a different manner of articulation at the same place, were defined as “substitution”. Errors in which the target consonant was replaced by an indeterminate sound outside the set of 21 consonants were defined as “distortion.” Errors involving deletion of the target consonant within a syllable were defined as “omission.” Errors in which an additional sound was inserted before or after the target consonant within a syllable were defined as “addition.”
(3) Classification of consonant error patterns: Based on manner-of-articulation deviations, error patterns were categorized as aspiration, deaspiration, fricativization, plosivization (stop formation), affricatization, lateralization, and nasalization. Based on place-of-articulation deviations, they were categorized as fronting, backing, retroflexion, and deretroflexion.

### 2.4. Short-Term Memory Assessment

The Digit Span subtest of the Wechsler Intelligence Scale for Children—Fourth Edition (WISC-IV) was administered. Scores and maximum span length were recorded for both forward and backward digit span. The maximum scores for forward and backward digit span are 18 and 14, respectively, yielding a total maximum score of 32. Forward digit span reflects short-term memory, whereas backward digit span reflects working memory.

### 2.5. Quality Control

Three physicians, each with at least 5 years of clinical experience in a speech–language outpatient setting, were involved in diagnosis, case enrollment, speech assessment, and speech error classification. Before the study, all physicians completed standardized training on the study protocol, administration procedures, and criteria for speech error identification and classification to ensure consistency across examiners. The structural integrity and basic motor function of speech-related organs (including the nose, pharynx, palate, and tongue), as well as audiological evaluations, were assessed by physicians with at least 5 years of experience in pediatric otorhinolaryngologic care. To ensure the accuracy of speech error classification, all speech recordings from all participants were independently reviewed and verified by a trained phonetician with at least 5 years of experience. The phonetician evaluated the consistency of transcription and error classification, and any discrepancies were resolved through discussion and consensus. Any discrepancies between the initial classification and the phonetician’s judgment were discussed and resolved through consensus. Children’s short-term memory was assessed by trained examiners who had received standardized instruction in the administration and scoring of the Digit Span subtest before data collection. All examiners underwent consistency training prior to formal testing, and their competency was monitored through periodic assessments during the study to ensure standardized administration and scoring.

### 2.6. Statistical Analysis

Statistical analyses were performed using SPSS version 21.0 and Prism 10. Normally distributed continuous variables are presented as mean ± standard deviation ($\bar{x}$ ± s). Between-group differences in consonant accuracy rates across age groups were evaluated using a one-way analysis of variance (one-way ANOVA). Between-group comparisons were performed using a two-way analysis of variance (two-way ANOVA). As all continuous variables met the assumption of normality, Pearson’s correlation analysis was used to examine bivariate associations. Linear regression analyses were subsequently conducted to identify factors predictive of consonant accuracy. All tests were two-sided, and a *p* value < 0.05 was considered statistically significant.

## 3. Results

### 3.1. Consonant Characteristics in School-Aged Children with SSD

The overall cohort (n = 142) demonstrated a mean accuracy of 0.72 ± 0.16, which corresponds to a mild-to-moderate severity level of speech impairment. Analysis across specific age subgroups revealed a non-linear developmental trend. While the consonant accuracy remained similar between the 6 to <7 years group (0.70 ± 0.16, n = 82) and the 7 to <8 years group (0.69 ± 0.17, n = 30), a notable increase was observed in the oldest group (≥8 years, 0.80 ± 0.11, n = 30). A one-way analysis of variance confirmed that these differences across age groups were statistically significant (F = 5.46, *p* = 0.005) (Table 1). In summary, the data indicate that consonant production accuracy in children with SSD is positively associated with age within the studied range, with a significant improvement evident in children aged 8 years and older, although all groups remained within the mild-to-moderate severity classification.

### 3.2. Error Types Across Consonants with Different Places of Articulation

Analysis of consonant error types by place of articulation revealed distinct profiles (Table 2). Consistent with the overall pattern, only substitution and omission errors were observed, whereas distortion and addition were not identified for any consonant. Substitution was the predominant error type for most consonants. Notably, among the apical posterior, the affricates [ʈʂ] (zh) and [ʈʂ^h^] (ch) showed the highest substitution rates (56.3% and 60.6%, respectively). Substitution also predominated among the labials, including [p^h^] (p) and [f] (f); apical anterior, including [ts] (z), [ts^h^] (c), and [s] (s); the velars, including [k] (g), [k^h^] (k), and [x] (h); and the alveolo-palatals, including [tɕ] (j), [tɕ^h^] (q), and [ɕ] (x). In contrast, omission was the primary error type for specific consonants, particularly [l] (l) among the apical medial (omission rate: 54.9%) and [ɻ] (r) among the apical posterior (omission rate: 15.5%). A subset of consonants showed high production accuracy (>90%), including the labials [p] (b) and [m] (m), the alveolars [t] (d) and [n] (n), and the alveolo-palatal affricate [tɕ] (j).

### 3.3. Error Patterns of Consonants with Different Places of Articulation

A notable finding was the frequent co-occurrence of multiple error patterns for a single consonant, as detailed in the table footnotes (Table 3). For example, plosivization often co-occurred with fronting or backing, whereas affricatization was frequently accompanied by backing. Each consonant showed a distinct error profile. Among the labials, [p^h^] (p) was characterized primarily by deaspiration and backing, whereas [f] (f) was mainly characterized by fronting and backing. Among the apical anterior, [ts] (z), [ts^h^] (c), and [s] (s) were predominantly characterized by plosivization, backing, and retroflexion. Among the apical medial, [t^h^] (t) was mainly characterized by backing, whereas [l] (l) was mainly characterized by aspiration and nasalization. Among the apical posterior, [ʈʂ] (zh) was mainly characterized by plosivization and fronting; [ʈʂ^h^] (ch) was mainly characterized by plosivization, fronting, backing, and deretroflexion; [ʂ] (sh) was predominantly characterized by plosivization, fronting, and deretroflexion; and [ɻ] (r) was mainly characterized by lateralization. Among the velars, [k] (g), [k^h^] (k), and [x] (h) were predominantly characterized by fronting. Among the alveolo-palatals, [ɕ] (x) was predominantly characterized by affricatization.

### 3.4. Speech and Short-Term Memory Profiles in School-Aged Children with SSD

**Figure 1:**
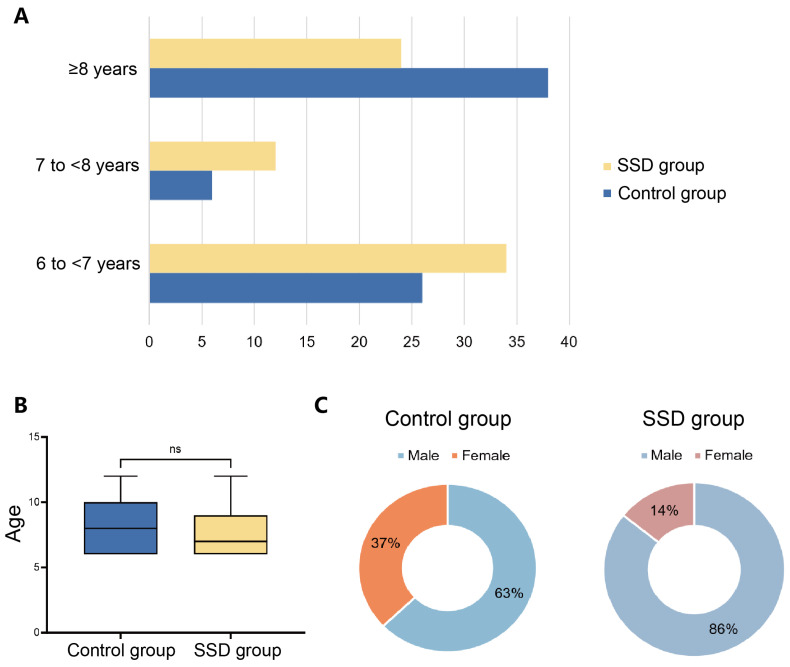
Demographic characteristics of participants. (**A**) Distribution of children across age strata (6 to <7 years, 7 to <8 years, and ≥8 years) in the SSD group and control group. Numbers at the end of each bar indicate participant counts within each stratum. (**B**) Box-and-whisker plots comparing chronological age between groups; the central line denotes the median, boxes indicate the interquartile range (IQR), and whiskers represent the range. (**C**) Sex distribution within each group shown as donut charts, with percentages of males and females indicated.

As shown in Figure 1, a total of 140 children were included, with 70 in the control group and 70 in the SSD group. Age-stratified distributions (Figure 1A) indicated that, in the SSD group, 34/70 were aged 6–<7 years, 12/70 were aged 7–<8 years, and 24/70 were ≥8 years, whereas the corresponding numbers in the control group were 26/70, 6/70, and 38/70, respectively. While the distribution of participants across age strata was not identical between the SSD and control groups, the boxplot comparison indicated no significant difference in chronological age between groups (Figure 1B). In contrast, the sex distribution differed between groups, with a higher proportion of males in the SSD group than in the control group (Figure 1C).

**Figure 2:**
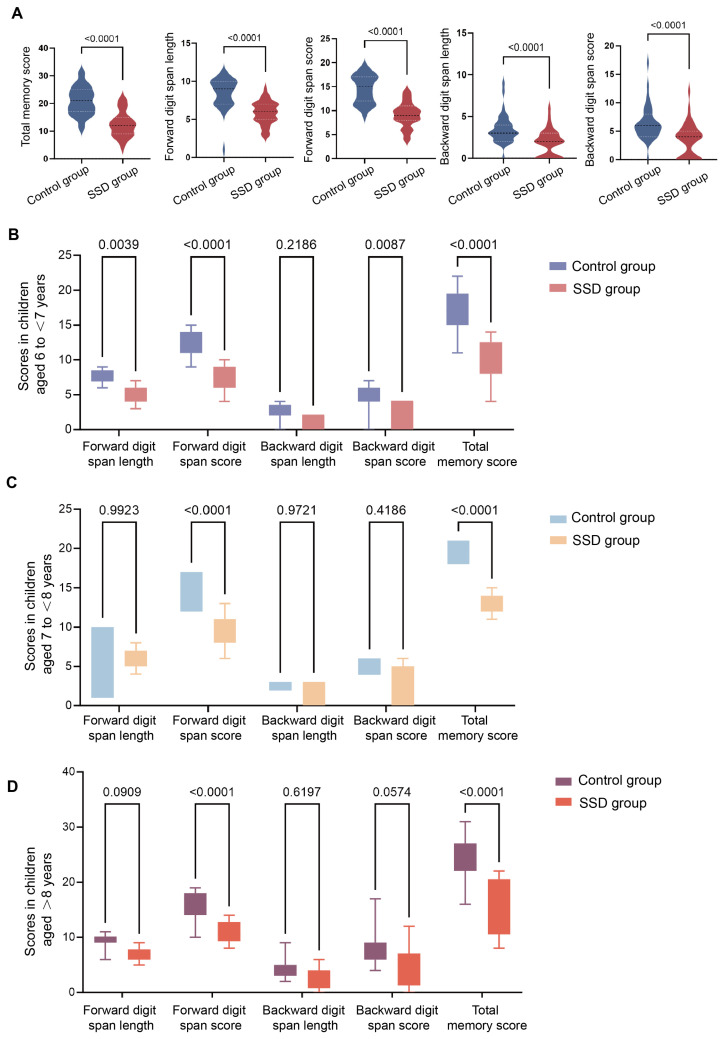
Group differences in digit span performance between children with SSD and control group, overall and stratified by age. (**A**) Violin plots comparing total memory score, forward digit span length, forward digit span score, backward digit span length, and backward digit span score between the control group and the SSD group. Horizontal dashed lines indicate central tendency and dispersion within each distribution. (**B**–**D**) Age-stratified comparisons for children aged 6 to <7 years (**B**), 7 to <8 years (**C**), and ≥8 years (**D**). Data are presented as box-and-whisker plots for each digit span index, with the center line denoting the median, boxes representing the interquartile range (IQR), and whiskers indicating the range. Between-group differences were evaluated using two-sided statistical tests; exact *p* values are displayed above brackets (with *p* < 0.0001 indicating strong significance).

As shown in Figure 2, compared with the control group, children with SSD exhibited significantly lower total memory score, forward digit span length, forward digit span score, backward digit span length, and backward digit span score (all *p* < 0.0001), indicating an overall impairment in short-term memory in the SSD group (Figure 2A). After age stratification (Figure 2B–D), in children aged 6 to <7 years, the SSD group showed reduced forward digit span length (*p* = 0.0039), forward digit span score (*p* < 0.0001), backward digit span score (*p* = 0.0087), and total memory score (*p* < 0.0001), whereas the difference in backward digit span length between two groups was not significant (*p* = 0.2186). In the 7 to <8 years subgroup, significant differences were observed only for forward digit span score (*p* < 0.0001) and total memory score (*p* < 0.0001), with no significant between-group differences for the remaining indices. Similarly, among children aged ≥8 years, the primary group differences were confined to forward digit span score (*p* < 0.0001) and total memory score (*p* < 0.0001), whereas the remaining measures did not reach statistical significance. Collectively, these findings suggest that SSD are associated with reduced speech-related short-term memory, with age-stratified analyses indicating that the most robust between-group differences are reflected in the forward span score and total memory score.

### 3.5. Correlation Between Consonant Accuracy and Short-Term Memory

**Figure 3:**
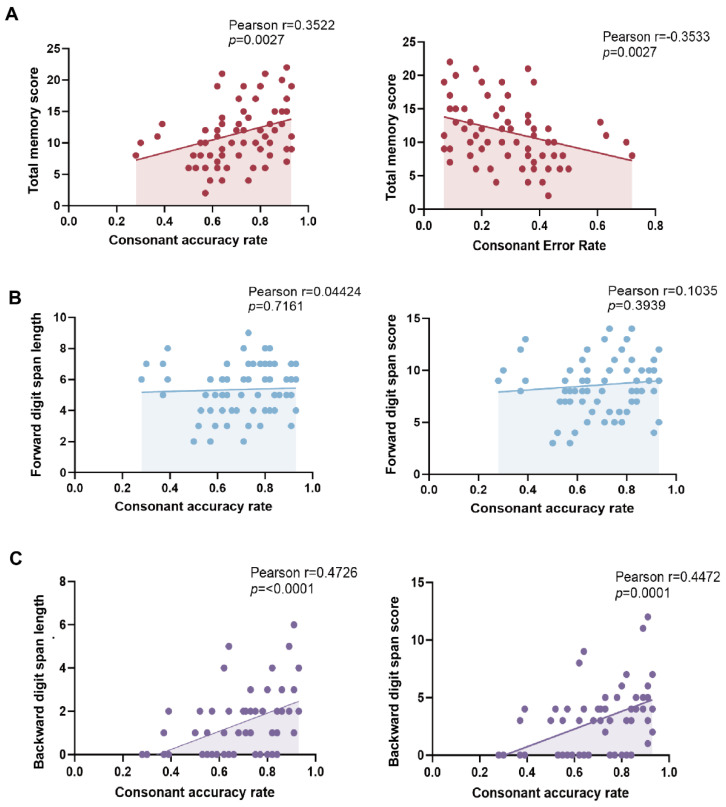
Associations between consonant production performance and short-term memory measures. (**A**) Correlation between total memory scores and consonant accuracy rate (left), consonant error rate. (**B**) Forward digit span length (left) and forward digit span score (right) in relation to consonant accuracy rate. (**C**) Backward digit span length (left) and backward digit span score (right) in relation to consonant accuracy rate. Each point represents one participant; solid lines indicate least-squares linear fits. Pearson correlation coefficients and corresponding two-tailed *p* values are shown in each panel.

Total memory scores were significantly positively correlated with consonant accuracy (Pearson’s r = 0.3522, *p* = 0.0027) and significantly negatively correlated with consonant error rates (r = −0.3533, *p* = 0.0027) (Figure 3A). After further decomposing the total memory scores into its subcomponents, neither forward digit span length (r = 0.04424, *p* = 0.7161) nor forward digit span score (r = 0.1035, *p* = 0.3939) showed a significant association with consonant accuracy. In contrast, backward digit span length (r = 0.4726, *p* < 0.0001) and backward digit span score (r = 0.4472, *p* = 0.0001) were both significantly positively correlated with consonant accuracy. Overall, consonant production ability is related to short-term memory, with the association predominantly driven by backward span indices.

### 3.6. Predictors of Consonant Accuracy in School-Aged Children with Speech Sound Disorders

The multiple linear regression model significantly predicted consonant accuracy in children with speech sound disorders. As shown in Table 4, the backward digit span score emerged as a significant positive predictor (β = 0.553, *p* = 0.009). In contrast, the total memory score did not make a significant independent contribution to the model (β = −0.119, *p* = 0.562).

## 4. Discussion

This study systematically examined consonant error profiles in school-aged Mandarin-speaking children with SSD and their association with short-term memory. We found that substitution errors were the most prevalent pattern across the majority of consonants, and that multiple error patterns frequently co-occurred for a given phoneme. Research on short-term memory in school-aged children with SSD further indicates that children with SSD performed significantly below their typically developing peers across multiple dimensions of short-term memory. The backward digit span score was significantly and positively correlated with consonant accuracy and emerged as an independent predictor of consonant accuracy. These findings indicate that, in school-aged children with SSD, difficulties are often not confined to “pure” articulatory impairment but may co-occur with broader, system-level vulnerabilities in language and speech (Tambyraja et al., 2023).

In our study, substitution errors emerged as the most prevalent error pattern across the majority of consonants; this result is in line with most Chinese research (Wang et al., 2020). These observations suggest that, for many children with SSD, the primary difficulty may lie less in random articulatory breakdown and more in imprecise or immature phonological representations and/or phonological planning, which then manifests as predictable sound replacements. However, the situation differs for the population of children diagnosed with speech sound disorders within English-speaking linguistic contexts. In Farquharson’s research, speech errors of English-speaking children with persistent SSD are frequently characterized by consistent, rule-governed substitutions of specific phonemes (/s/→/θ/) (Farquharson et al., 2018). Nevertheless, research by Preston and Edwards on children with speech sound disorders indicated that their speech errors consist primarily of distortions, substitutions, and omissions, with distortions being the most prevalent (J. Preston & Edwards, 2010). Furthermore, studies have also shown that among English-speaking children with SSD, initial syllable deletion is the most frequent error pattern observed during testing at the single-word level (Brosseau-Lapré & Roepke, 2019; Hua & Dodd, 2000). These findings suggest that the primary patterns of consonant errors differ across languages in children with speech sound disorders. This understanding of error patterns by place of articulation, when integrated with targeted intervention strategies, enables more tailored and effective speech training.

According to the developmental trajectory of Putonghua phonemes, 75% of children acquire the 21 consonants by the age of 3, and by 4.5 years of age, 90% of children can clearly articulate words beginning with these 21 consonants. Between the ages of 1.5 and 4.5, children progressively master bilabial, laminal, and velar sounds, with apical sounds being acquired last (Hua & Dodd, 2000). Some consonant errors may persist into school age and therefore merit attention. In this study, the highest error rates were observed for velar and apical consonants. As the articulatory difficulty of consonants increased, the proportion of children with consonant errors also rose, which is consistent with our previous research findings that retroflex consonants have the highest error rate in children with functional speech sound disorders, and that some of these errors do not fully resolve with age (Wang et al., 2020). This indicates that in typical development, speech sounds that are acquired later, mature later, and are more difficult are more prone to errors. This finding further supports the view proposed by Preston et al. (J. L. Preston et al., 2013) and Wren et al. (Wren et al., 2016) that speech sound problems in school-age children are a continuation of those present during the preschool period. Therefore, attention should be given to the acquisition of apical and velar consonants, and early intervention guidance should be provided. For children with speech sound disorders, speech training goals can be established by taking the speech sound development sequence observed in typical development as a reference.

Our results also show consonant error patterns in children with SSD clustered in later-acquired, high-precision segments—particularly sibilant fricatives/affricates, retroflex sibilants, and velars. The dominant pattern was manner simplification, including [ts] (z), [ts^h^] (c), [ʈʂ] (zh), [ʈʂ^h^] (ch), and [ʂ] (sh), suggesting a preference for complete oral closure over the fine aerodynamic control required for sustained fricativization. In parallel, place contrasts were unstable, alveolar sibilants often shifted posteriorly (including retroflexion/backing), whereas retroflex targets frequently showed the opposite direction (deretroflexion/fronting), and velars were commonly fronted, together indicating blurred category boundaries. This interpretation aligns with scoping evidence in SSD showing that more deviant or atypical error profiles are linked to greater persistence into school age and increased vulnerability in phonological–literacy domains (Roepke, 2025). Collectively, our findings underscore the need for intervention that targets both fricativization control and stable place contrasts.

Our findings indicate a generalized reduction in STM capacity in school-aged children with SSD. At the group level, the most robust differences between children with SSD and typically developing controls were observed for forward digit span performance, whereas within the SSD group, backward digit span was more closely associated with consonant accuracy. These results are consistent with previous research and support the view that SSD is not solely a speech-motor or articulatory difficulty, but may involve broader limitations in the temporary storage mechanisms that support speech processing and learning (Berti et al., 2022; Torrington Eaton & Ratner, 2016). Forward digit span primarily indexes verbal STM storage capacity and the integrity of phonological representations, with comparatively lower demands on executive manipulation than backward span (Giofrè et al., 2016; Monaco et al., 2013; Pham & Archibald, 2023). Therefore, the stronger group-level difference in forward digit span may indicate that reduced phonological storage capacity is a broader and more common characteristic of SSD.

Importantly, between-group comparisons and within-group association analyses address different questions. The former examines whether children with SSD, as a group, differ from typically developing peers, whereas the latter examines which cognitive factors account for variability in speech performance among children who already have SSD. In this context, backward digit span may not function as the most sensitive marker for distinguishing SSD from typical development at the group level, but it may be more sensitive to the degree of cognitive–linguistic burden associated with consonant production within the SSD group itself. Compared with findings more commonly emphasized in the English-language literature, our results showed that backward digit span was independently associated with speech accuracy in Mandarin-speaking children. The backward digit span task places greater demands on the central executive component of verbal working memory, requiring not only temporary retention but also active manipulation and reordering of verbal material (Füllgrabe & Öztürk, 2022; Kessels et al., 2008). Its association with speech accuracy suggests that consonant production difficulties may reflect not only articulatory limitations but also higher-order cognitive–linguistic constraints. From a clinical perspective, this result indicates that the assessment of children with SSD may benefit from including measures of verbal working memory in addition to speech production tasks alone. It also suggests that intervention may need to consider cognitive–linguistic support, particularly for children whose consonant production difficulties co-occur with weaknesses in verbal information processing. To our knowledge, comparable evidence from non-English-speaking populations remains limited. Therefore, the present findings should be interpreted cautiously, while also highlighting the need for further cross-linguistic research on the relationship between speech production and verbal working memory.

This study delineated the most prevalent consonant error patterns in children with SSD and highlighted the contributory role of working memory in speech production. These findings support incorporating short-term memory screening into routine SSD assessments to better characterize cognitive–linguistic profiles and identify children who may be at elevated risk for persistent speech difficulties. Moreover, intervention programs may benefit from integrating speech-related short-term memory training alongside conventional speech therapy.

There are several limitations to this study. First, the sample comprised only Mandarin-speaking children; therefore, caution is warranted when generalizing these findings to other linguistic populations. Second, we did not fully control for potential confounding factors, such as the home language environment, which may influence both speech outcomes and cognitive performance. Third, the assessment of short-term memory relied on a relatively limited set of measures. In addition, we acknowledge that the ≥8 years group covered a relatively broad age range compared with the two younger groups. Although this grouping was adopted for developmental and sample-size considerations, the heterogeneity within this group may have reduced the precision of age-group comparisons. Therefore, findings involving the ≥8 years group should be interpreted with caution, and future studies with larger samples should consider using narrower age bands in older children. We also acknowledge that the SSD and control groups were not matched exactly on age strata and sex. Although chronological age did not differ significantly between groups, the distribution across age categories was not identical, and the SSD group included a higher proportion of males. These sample characteristics may have increased the difficulty of between-group comparisons and should be taken into account when interpreting the findings.

Future research should pursue longitudinal designs to delineate the dynamic, developmental relationship between short-term memory and speech acquisition over time. In addition, it will be important to develop and rigorously evaluate speech intervention programs that explicitly integrate short-term memory training. Finally, cross-linguistic comparative studies are needed to test the generalizability of the present findings and to clarify the extent to which language-specific phonological properties modulate the association between short-term memory and speech accuracy.

In summary, this study demonstrates that Mandarin-speaking school-aged children with SSD exhibit marked deficits in both consonant production and speech-related short-term memory. Importantly, we provide the first evidence that backward digit span score serves as a key cognitive indicator predicting speech accuracy in this population. These findings not only deepen our understanding of the mechanisms underlying SSD but also offer a scientific rationale for developing future intervention approaches that integrate cognitive training with speech-focused therapy to optimize clinical outcomes.

## Figures and Tables

**Table 1:** Consonant accuracy rate in children with speech sound disorders (mean ± SD).

Group (Age)	n	Consonant Accuracy Rate (Mean ± SD)	Severity Level
6 to <7 years	82	0.70 ± 0.16	Mild-to-moderate
7 to <8 years	30	0.69 ± 0.17	Mild-to-moderate
≥8 years	30	0.80 ± 0.11	Mild-to-moderate
Total	142	0.72 ± 0.16	Mild-to-moderate
F = 5.46; *p* = 0.005			

**Table 2:** Error types of consonants by place of articulation [n (%)].

**Error Type**	**Labials**				**Apical Anterior**			**Apical Medial**					
	**p**	**p^h^**	**m**	**f**	**ts**	**ts^h^**	**s**	**t**	**t^h^**		**n**		**l**
Correct	136 (95.8)	122 (85.9)	140 (98.6)	106 (74.6)	58 (40.8)	54 (38.0)	76 (53.5)	134 (94.4)	116 (81.7)		136 (95.8)		48 (33.8)
Substitution	6 (4.2)	16 (11.3)	2 (1.4)	32 (22.5)	78 (54.9)	84 (59.2)	62 (43.7)	8 (5.6)	24 (16.9)		2 (1.4)		16 (11.3)
Omission	0 (0)	4 (2.8)	0 (0)	4 (2.8)	6 (4.2)	4 (2.8)	4 (2.8)	0 (0)	2 (1.4)		4 (2.8)		78 (54.9)
Distortion	0 (0)	0 (0)	0 (0)	0 (0)	0 (0)	0 (0)	0 (0)	0 (0)	0 (0)		0 (0)		0 (0)
Addition	0 (0)	0 (0)	0 (0)	0 (0)	0 (0)	0 (0)	0 (0)	0 (0)	0 (0)		0 (0)		0 (0)
**Error Type**	**Apical Posterior**				**Velars**			**Alveolo-Palatals**					
	**ʈʂ**	**ʈʂ^h^**	**ʂ**	**ɻ**	**k**	**k^h^**	**x**	**tɕ**		**tɕ^h^**		**ɕ**	
Correct	62 (43.7)	56 (39.4)	72 (50.7)	102 (71.8)	80 (56.3)	82 (57.7)	122 (85.9)	128 (90.1)		122 (85.9)		116 (81.7)	
Substitution	80 (56.3)	86 (60.6)	62 (43.7)	18 (12.7)	58 (40.8)	58 (40.8)	14 (9.9)	12 (8.5)		18 (12.7)		22 (15.5)	
Omission	0 (0)	0 (0)	8 (5.6)	22 (15.5)	4 (2.8)	2 (1.4)	6 (4.2)	2 (1.4)		2 (1.4)		4 (2.8)	
Distortion	0 (0)	0 (0)	0 (0)	0 (0)	0 (0)	0 (0)	0 (0)	0 (0)		0 (0)		0 (0)	
Addition	0 (0)	0 (0)	0 (0)	0 (0)	0 (0)	0 (0)	0 (0)	0 (0)		0 (0)		0 (0)	

**Table 3:** Error patterns of consonants across places of articulation [n (%)].

**Error Pattern**	**Labials**				**Apical Anterior**			**Apical Medial**					
	**p**	**p^h^**	**m**	**f**	**ts**	**ts^h^**	**s**	**t**	**t^h^**		**n**		**l**
Aspiration	-	-	-	-	-	-	-	-	-		-		16 (11.3)
Deaspiration	-	8 (5.6)	-	-	-	4 ^&^ (2.8)	4 ^&^ (2.8)	-	6 (4.2)		-		-
Fricativization	-	2 * (1.4)	-	-	-	-	-	-	4 * (2.8)		-		-
Plosivization	-	-	-	6 ^#^ (4.2)	32 (54.9)	46 *** (32.4)	12 *** (8.5)	-	-		-		-
Affricatization	-	-	-	2 ** (1.4)	-	-	2 (1.4)	-	-		-		-
Lateralization	-	-	-	-	-	-	2 (1.4)	-	2 (1.4)				-
Nasalization	-	-	-	-		-	-	-	-		-		16 (11.3)
Fronting	-	-	-	12 (8.5)	8 ^#^ (5.6)	-	4 (2.8)	-	-		-		-
Backing	6 (4.2)	8 (5.6)	2 (1.4)	18 (12.7)	24 *** (16.9)	50 (35.2)	16 (11.3)	8 (5.6)	20 (14.1)		-		-
Retroflexion	-	-	-	-	46 (32.4)	34 (23.9)	38 (26.8)	-	-		-		-
Deretroflexion	-	-	-	-	-	-	-	-	-		-		-
**Error Pattern**	**Apical Posterior**				**Velars**			**Alveolo-Palatals**					
	**ʈʂ**	**ʈʂ^h^**	**ʂ**	**ɻ**	**k**	**k^h^**	**x**	**tɕ**		**tɕ^h^**		**ɕ**	
Aspiration	6 ^##^ (4.2)	-	-	-	-	-	-	2 (1.4)		-		-	
Deaspiration	-	2 (1.4)	-	-	-	10 ^###^ (7.0)	-	-		6 (4.2)		-	
Fricativization	-	2 (1.4)	-	-	-	2 (1.4)	-	8 (5.6)		8 (5.6)		-	
Plosivization	40 ^#^ + 8 *** (29.6)	42 (28.2)	20 ^#^ (14.1)	-	-	-	-	-		4 ^#^ (2.8)		-	
Affricatization	-	-	8 (5.6)	-	-	-	-	-		-		22 (15.5)	
Lateralization	-	-	-	18 (12.7)	-	-	-	-		-		-	
Nasalization	-	-	-	-	-	-	-	-		-		-	
Fronting	46 (32.4)	28 ^#^ (19.7)	24 (16.9)	-	58 (40.8)	56 (39.4)	14 (9.9)	2 (1.4)		4 (2.8)		-	
Backing	8 (5.6)	14 *** (9.9)	-	-	-	-	-	-		-		-	
Retroflexion	-	-	-	-	-	-	-	-		-		-	
Deretroflexion	26 (18.3)	40 (28.2)	30 (21.1)	-	-	-	-	-		-		-	

* Fricativization co-occurred with backing; ** Affricatization co-occurred with backing; *** Plosivization co-occurred with backing; ^#^ Plosivization co-occurred with fronting; ^##^ Plosivization and aspiration co-occurred with fronting; ^###^ Deaspiration co-occurred with fronting; ^&^ Plosivization, deaspiration, and backing co-occurred.

**Table 4:** Multiple Linear Regression Analysis Predicting Consonant Accuracy.

Predictor	β	*p*	95% CI for *B*
Backward span score	0.553	0.009	(0.008, 0.052)
Total memory score	−0.119	0.562	(−0.018, 0.010)

## Data Availability

The original contributions of this study are provided in the article. Further inquiries may be addressed to the corresponding authors.
